# Stereotactic ablative radiotherapy for inoperable T1‐2N0M0 small‐cell lung cancer

**DOI:** 10.1111/1759-7714.14355

**Published:** 2022-02-17

**Authors:** Hui Tang, Wei Liu, Kaiming Huang

**Affiliations:** ^1^ Department of Intervention Lishui People's Hospital, 6th Affiliated Hospital of Wenzhou Medical University Lishui China; ^2^ Department of Thyroid Gland and Breast Surgery Lishui People's Hospital, 6th Affiliated Hospital of Wenzhou Medical University Lishui China

To the editor

Carcinoma of the lung is the leading cause of cancer‐associated death and the second most common carcinoma following breast cancer worldwide according to global cancer statistics for 2020.^1^ It is reported that 2 206 771 new lung cancer cases and 1 796 144 lung cancer deaths were estimated globally in 2020.^1^ Lung cancer is the first cause of cancer‐related death followed by colorectal cancer.^2^ Small‐cell lung cancer (SCLC), one of the two main types of lung carcinoma, accounts for 15%–20% of all lung cancers and has poor prognosis due to advanced stages when first diagnosis.^3^ However, about 5% of SCLC cases were at early stages (T1‐2N0M0) when first diagnosed, which may be a result of lung cancer early screening projects for high‐risk individuals and routine physical examination. Because of the potential to cure early stages of SCLC (T1‐2N0M0), operations such as lobectomy and mediastinal lymph nodes sampling are recommended according to the current SCLC treatment guidelines for operable cases. For inoperable cases, concurrent chemoradiation is recommended according to literature. However, the toxicity of the concurrent chemoradiation modality is severe and risk of grade 3–5 treatment‐related toxicity is high.

Recent studies have indicated that stereotactic ablative radiotherapy (SABR) is not inferior to conventional radiation in the aspects of local control or survival, and is even comparable to operation for early‐stage non‐small‐cell lung cancer (NSCLC).^4^ SABR in the treatment of early stage (T1‐2N0M0) SCLC has been also investigated in recent clinical studies, has demonstrated satisfactory outcomes, and is recommended by American Society for Radiation Oncology (ASTRO) Clinical Practice Guidelines.^5^ However, the ASTRO guidelines were based on single‐arm small sample size clinical studies with limited statistical power and weak evidence. Safavi and his colleagues^6^ have therefore performed a comprehensive meta‐analysis relevant to SABR in T1‐2N0M0 SCLC by combining 11 clinical studies, including 399 early‐stage cases, on aspects of local control, overall survival, 1‐year survival, 2‐year survival, recurrence and treatment toxicity. The authors found that SABR for inoperable early‐stage, node‐negative SCLC was effective for local control and the treatment toxicity was limited and acceptable. The statistical power of SABR for early‐stage SCLC was strengthen by combination 11 associated studies. The evidence level may also be elevated for SABR in the treatment of early‐stage SCLC by Safavi's work. However, there were also limitations for the meta‐analysis: (i) the studies included in the meta‐analysis were nonrandomized single‐arm studies without control groups, therefore whether or not SABR was superior to other treatment modalities such as concurrent chemoradiation was not clear; (ii) most of the studies included in the meta‐analysis were retrospective and were not of high methodical quality; (iii) clinical heterogeneity across the 11 studies was obvious such as without pathological confirmation, PCI application, previously treatment modality and etc., which may decrease the evidence level; and (iv) the sample size of three included studies was too small (only eight and six cases included), which may had limited statistical power, and only one study had large sample size (*n* = 239), but without pathological confirmation of SCLC. Because of these limitations, whether or not SABR is the best treatment choice for early‐stage inoperable SCLC is not yet completely clear. Further well‐designed prospective randomized clinical trials comparing SABR to other treatment modalities in the outcomes of local control, long‐term survival, and treatment‐related toxicities are therefore needed to further evaluate the role of SABR for cases of early‐stage (T1‐2N0M0) inoperable SCLC.^7^

## CONFLICT OF INTEREST

The authors declare that the research was conducted in the absence of any commercial or financial relationships that could be construed as a potential conflict of interest.
